# Book Reviews

**Published:** 1894-06

**Authors:** 


### Book Reviews.

A PRACTICAL TREATISE ON MEDICAL DIAGNOSIS, for Students and Physicians. By JOHN H. MUSSER, M. D., Assistant Professor of Clinical Medicine in the University of Pennsylvania, Philadelphia; President of the Pathological Society of Philadelphia. Octavo, 873 pages, 162 engravings and two colored plates. Cloth, $5.00; leather, $6 00. Philadelphia: Lea Brothers & Co. 1894.

The importance of a thorough training in medical diagnosis is everywhere recognized as a necessary foundation to the intelligent application of the principles and details of treatment. Diagnosis is a neglected part of medical science—neglected by teachers, neglected by students. For a long time Da Costa's classic work held sway in this field, and was almost the only text-book on medical diagnosis referred to in college announcements. Of late years, however, several treatises on the subject have appeared, and we feel sure that Musser's work will take rank with the best of these.

The author divides his subject into two parts, namely—general diagnosis and special diagnosis. General diagnosis is treated of in Part I., comprising the first six chapters of the book. Special diagnosis forms the subject matter of Part II., comprising eleven chapters. Under general diagnosis are considered general observations, the data obtained by inquiry, the data obtained by observation, bacteriological diagnosis, the examination of exudations, transudations and cystic fluids and the morbid processes and their symptomatology. A new impetus has been given to medical diagnosis by modern instruments and bacteriological research, hence the chapter on bacteriological diagnosis will challenge the attention of all who place reliance on that important collateral science as a factor in placing the determination of disease on a basis of scientific certainty. The photograph, too, is made to play an important part in the diagnosis of certain diseases, especially those of a neurotic origin and in deformities due to structural change.

In the second part of the book the various regions of the body are considered and made to tell their several tales with reference to morbid processes that may have seized upon them. Finally, constitutional diseases, the infectious diseases and diseases of the nervous system are considered in the order of sequence named. An excellent index is added to facilitate reference. This author's experience as a teacher is such as to attract attention to his work at once, and a careful examination of his book will convince anyone familiar with the subject that its excellent qualities are many, that its defects are few and that it must take rank as one of the best American treatises on the subject, and that, besides, it has no superior in any tongue or country.

---

Lectures on Auto-Intoxication in Disease, or Self-Poisoning of the Individual. By Ch. Bouchard, Professor of Pathology and Therapeutics, Member of the Academy of Medicine, and Physician to the Hospitals, Paris. Translated, with a preface, by Thomas Oliver, M. A., M. D., F. R. C. P., Professor of Physiology, University of Durham; Physician to the Royal Infirmary, Newcastle-upon Tyne; and Examiner in Physiology, Conjoint Board of England. In one octavo volume; 302 pages. Extra cloth, $1.75 net. Philadelphia: The F. A. Davis Co., Publishers, 1914 and 1916 Cherry street. 1894.

The author of this work begins with an assertion that has a volume of common sense in it. He says that medicine, after having devoted herself for many years to the verification of symptoms, to the research of anatomical lesions, and to the study of pathological physiology, comes finally to the study of the origin of disease. It is only lately that physicians have begun to recognize that putrefactive processes in the intestinal canal, and the development of physiological and pathological alkaloids, play an important part in causing many diseases, the origin of which has been hitherto little understood or greatly misunderstood. How often it happens that comparatively sudden death occurs in individuals in apparent good health, where carefully conducted autopsies fail to reveal lesions that account for the fatality. In many such cases, no doubt, toxines enter the blood, and pervert or destroy its nutritive qualities.

Bouchard has covered a great field in these lectures, and they should be carefully studied by every physician. Here he will find explained the causes of the toxicity of urine, and the part toxic principles play in producing uremia. So, too, are discussed the toxicity of the feces, and an entire lecture is devoted to intestinal autisepsis. One of the most interesting parts of the work is that where the author discusses the relation of auto-intoxication to typhoid fever. The pathogenic therapeusis of typhoid fever, the antisepsis of the intestinal canal, the treatment of high temperature, modes of bathing in fevers and the dieting of fever patients, receive most elaborate and careful handling. If physicians would study this kind of literature more, they would better fit themselves to cope with typhoid disease than when cursorily examining a book of formulæ for ready-made prescriptions.

The pathogenesis of jaundice receives detailed and finished attention, and the method of auto-intoxication by bile is explained. Cholera forms the subject of three lectures, which is taken as a type of those diseases caused by poisoning, though Bouchard looks upon it as an infectious disease, but doubts whether Koch's pathogenic agent is the true one. He believes that the cholera poison is eliminated by the urine, but has not been able to make cultures of the urine of cholera patients.

Not the least interesting portion of this book is the preface by the translator, and it also possesses a carefully prepared index. This is one of the modern classics of medical literature and will easily find its way into the hands of every studious physician.

A MANUAL OF NURSING IN PELVIC SURGERY. By LEWIS S. MCMURTRY, A. M., M. D., Professor of Gynecology in the Hospital College of Medicine, Louisville; Surgeon-in-Charge of the Jennie Cassady Infirmary for Women; Gynecologist to Sts. Mary and Elizabeth Hospital, etc. Duodecimo, pp. 92. Morton's Pocket Series, No. 3. Louisville: John P. Morton & Company. 1894.

Though a number of books have been published giving instructions to nurses on general as well as special departments of their profession, this is the first that has been issued with the special object of giving instructions on the duty of nurses with reference to surgical operations exclusively in the pelvic cavity.

The distinguished author of this little treatise was one of the first surgeons in America to differentiate pelvic from abdominal surgery, and to classify operative gynecology under the latter head. This treatise has this differentiation as an underlying principle running all through it, which makes it distinctive, original and unique. As might be expected, it betrays the master's hand in every page and paragraph, and is one of the most concise, instructive and useful manuals that has been issued, and, so far as we know, is the only one especially adapted to nursing in pelvic surgery.

It should be found in every nurse's hands who essays to do such work, and, if followed, will constitute a safe guide in every detail. One aphorism the author quotes from Dr. Weir Mitchell, deserves to be written as a motto over every book on nursing, and we cannot resist the temptation to reproduce it here. Mitchell gives as prime qualifications, "good breeding and loveliness of manners;" and another from Mr. Greig Smith, also quoted by McMurtry, deserves to be placed alongside of it. Smith in his classic treatise on abdominal surgery says: "A perfect nurse is a perfect woman," and he mentions among her most essential characteristics, "good temper, gentleness and cleanliness." To these qualities McMurtry would add, "a keen sense of personal responsibility and conscientious appreciation of duty," to all of which we further add our most hearty approbation.

---

THE JOHNS HOPKINS HOSPITAL REPORTS. Report in Gynecology, II. Volume III.; Nos. 7, 8, 9. Imperial octavo, pp. 461. Illustrated. Baltimore: The Johns Hopkins Press. 1894.

This book is mainly the record of the work of Dr. H. A. Kelly, professor of gynecology in Johns Hopkins University. Some of the articles are especially interesting, by reason of the rare conditions that they record. As such we may mention one on prolapsus uteri, without vesical diverticulum and without anterior enterocele; and another on lipoma of the labium majus. Dr. Kelly has grouped twenty such cases that he has found in medical literature, one of which was his own. This occurred in a negress, aged twenty-seven years, occupied the right labium majus, in size 2 x 1½ x 1 cm., and was cured by extirpation.

Dr. Kelly is a believer in operations for the suspension of the retroflexed uterus, and abandons the name hysterorrhaphy that he first gave to this operation, objects to the terms ventrofixation and hysteropexy and adopts the term suspensio uteri as being more descriptive. He gives a table of forty-five cases, which all recovered from the primary operation, and describes in detail their subsequent condition. One of the most interesting chapters in the book treats upon some sources of hemorrhage in abdominal pelvic operations, but this might be considerably enlarged with benefit.

Photography applied to surgery is an interesting subject discoursed upon by A. S. Murray. Resuscitation in chloroform asphyxia is an absorbing subject, but is also too briefly handled by Dr. Kelly. A very important part of this report is a record of deaths without operation, in which detailed autopsies are given, showing pathological findings and demonstrating, in many instances, the propriety of early operation as a life-saving means.

The work is copiously illustrated and contains many elaborate tables of a statistical character. The price is $3.00, and can be obtained from the Johns Hopkins Press, Baltimore.

---

The Physician's Wife, and the Things that Pertain to Her Life. By Ellen M. Firebaugh. With portrait of author and forty-four photo-engravings of original sketches. In one crown octavo volume of 200 pages. Extra cloth, $1.25 net. Special limited edition, first 500 copies numbered, and printed in photogravure ink, on extra fine enameled paper; bound in half-leather and vellum cloth, $3.00 net. Philadelphia: The F. A. Davis Co., Publishers, 1914 and 1916 Cherry street. 1894.

While this book can hardly be said to be a contribution to medical literature, it may serve as a light diversion to the practitioner from his more serious and too often wearying anxieties and studies. It is true, the work cannot be classed among the models of literary skill or artistic finish, but there is a breezy earnestness about it that makes it far from commonplace, and we may be sure that it comes from a head and a heart that are both where they ought to be. It is curious to note the different view of life taken by the successful doctor's wife, whose experience is limited to a more or less bucolic environment from that of the city physician's helpmate. The amusing anecdotes and clever illustrations, with which the book abounds, all trend in one direction—namely, to show that a doctor's life is a hard one, and that his wife's is harder still. We cannot but be amused at the reasons given for the necessity of not calling another physician in case of her own illness, though she may very much desire to have one—namely, that it would injure her husband's practice!

Taken all in all, we can heartily recommend Mrs. Firebaugh's book as a pleasant, spicy and amusing production, likely to be highly enjoyed by her for whom it seems to be especially written —the country physician's wife.

---

Syllabus of Lectures on the Practice of Surgery, arranged in conformity with the American Text-book of Surgery. By N. Senn, M. D., Ph. D., LL. D., Chicago, Professor of the Practice of Surgery and Clinical Surgery in Rush Medical College; Professor of Surgery in the Chicago Polyclinic; Attending Surgeon to Presbyterian Hospital; Surgeon-in-Chief St. Joseph's Hospital, etc., etc. Philadelphia: W. B. Saunders, 295 Walnut street. 1894.

A two-fold aim actuated the author in preparing his syllabus of surgery. In the first place, the work was designed to assist teachers of surgery, by placing at their command a systematic and condensed presentation of the various subjects included in a surgical course. In the second place, the work was to aid the student in his note-taking and memorizing of facts, and the author has endeavored to assist him in this by giving subjects their relative place in the scale of importance and worth. In the main the aim of the book has been accomplished, and it can be thoroughly recommended to teachers and students, who will find it most useful to them. J. P.

---

Operative Surgery. By Th. Kocher, M. D., Professor at the University and Director of the Surgical Clinic at the Berne University. Octavo, 288 pages, 163 illustrations. Extra muslin, price, $3.00. New York: William Wood & Company. 1894.

The object of this work is to assist surgeons in acquiring a mastery of operative technique. The modern antiseptic treatment of wounds has so materially modified operative technique that it has been necessary to rewrite much of the surgery that stood in the pre-Listerean period ; either this or to supplement standard works by a working manual devoted specially to technique, like the book under consideration.

The personal equation enters largely into this treatise, since Kocher describes and advises only those methods that he has thoroughly and practically tested. It, therefore, becomes a very readable book even if, in some instances, his opinions are not accepted. One of its chief advantages is, that the surgeon may quickly inform himself on a particular point without reading through a mass of literature that would consume much time. The illustrations are well drawn in many instances, but there is here yet room for improvement. This is especially the case with reference to illustrations bearing on regional surgery. It goes without saying that every surgeon will make haste to possess this work.

---

VENEREAL MEMORANDA. A Manual for the Student and Practitioner. By P. A. MORROW, A. M., M. D., Clinical Professor of Venereal Diseases in the University of the City of New York ; Surgeon to Charity Hospital ; Attending Surgeon to the Bellevue Hospital Out-Door Relief, Department of Skin Diseases ; Member of the American Dermatological Association ; Member of the New York Dermatological Society, etc., etc. Double duodecimo, pp. iv.—332. Second edition. New York : William Wood & Co. 1894.

The object of this work is to present to the profession a concise exposition of the nature and treatment of venereal diseases. The first edition, issued in 1886, was exhausted some time ago, and this second edition has been delayed to enable the author to make such changes as would bring the book fully up to date. It is a convenient reminder of methods of treatment to be employed in venereal diseases, but is not intended to supplant more elaborate treatises.

---

PROCEEDINGS OF THE PHILADELPHIA COUNTY MEDICAL SOCIETY. Volume XIV. Session of 1893. LEWIS H. ADLER, JR., M. D., Editor. Octavo, pp. xxviii.—484. Philadelphia : William J. Dornan. 1893.

The annual volume issued by this society is always full of excellent literature. In fact, the Philadelphia County Medical Society is one of the most active and best disciplined county societies in the country. Many of the papers contained in this book have been published in the medical journals, but it is always well to have society proceedings bound in annual volumes for reference. Once let a society fail to do this and its literature is scattered to the four winds, its usefulness is detracted from and its cohesiveness impaired. Such volumes as this should be found in every medical library.

---

A Manual of Minor Surgery and Bandaging, for the Use of House-Surgeons, Dressers and Junior Practitioners. By Christopher Heath, F. R. C. S., Surgeon to University College Hospital and Holme Professor of Clinical Surgery in University College, London ; Member of the Council of the Royal College of Surgeons of England. Tenth edition. Illustrated, 16mo, pp. xvi.—389. Price, $2.00. Philadelphia : P. Blakiston, Son & Co., 1012 Walnut street. 1894.

This little work is an old friend and needs very little further notice at this time beyond the mere announcement that the tenth edition is now ready on the shelves of the book stores for purchasers. In this edition the distinguished author has embodied the latest teachings regarding antiseptics, and in various other ways has modernized it to keep pace with the progress that the newer surgery is making. It is intended as a manual for the guidance of those who are constantly employed as assistants, and it would also be a useful book for nurses.

---

### BOOKS RECEIVED.

Essentials of Anatomy, Including the Anatomy of the Viscera. Arranged in the form of Questions and Answers ; prepared especially for Students of Medicine. By Charles B. Nancrede, M. D., Professor of Surgery and of Clinical Surgery in the University of Michigan, Ann Arbor ; Corresponding Member of the Royal Academy of Medicine, Rome, Italy, etc., etc. Saunders' Question Compends, No. 3. Fifth edition, with an appendix and 180 illustrations. Duodecimo, pp. x.—388. Price, $1.00. Philadelphia : W. B. Saunders, 925 Walnut street. 1894.

Essentials of Nervous Diseases and Insanity. Their Symptoms and Treatment. By John C. Shaw, M. D., Clinical Professor of Diseases of the Mind and Nervous System, Long Island College Hospital Medical School ; Consulting Neurologist to St. Catharine's Hospital and Long Island College Hospital. Saunders' Question Compends, No. 21. Second edition. Crown 8vo, 186 pages. Forty-eight original illustrations, mostly selected from the author's private practice. Price, cloth, $1.00 ; interleaved for notes, $1.25. Philadelphia : W. B. Saunders, 925 Walnut street. 1894.

International Clinics. A Quarterly of Clinical Lectures on Medicine, Neurology, Pediatrics, Surgery, Genito-Urinary Surgery, Gynecology, Ophthalmology, Laryngology, Otology and Dermatology. By professors and lecturers in the leading medical colleges of the United States, Great Britain and Canada. Edited by Judson Daland, M. D.,

Philadelphia, Instructor in Clinical Medicine and Lecturer on Physical Diagnosis in the University of Pennsylvania ; Assistant Physician to the University Hospital ; Physician to the Philadelphia Hospital and to the Rush Hospital for Consumption. J. Mitchell Bruce, M. D., F. R. C. P., London, England; Physician and Lecturer on Therapeutics at the Charing Cross Hospital. David W. Finlay, M. D., F. R. C. P., Aberdeen, Scotland, Professor of Practice of Medicine in the University of Aberdeen ; Physician to, and Lecturer on, Clinical Medicine in the Aberdeen Royal Infirmary ; Consulting Physician to the Royal Hospital for Diseases of the Chest, London. Volume I. Fourth series. 1894. Royal 8vo, pp. xii.—363. Philadelphia : J. B. Lippincott Co. 1894.

Diseases of the Skin ; An Outline of the Principles and Practice of Dermatology. By Malcolm Morris, F. R. C. S., Surgeon to the Skin Department, St. Mary's Hospital, London, etc. In one 12mo volume of 572 pages, with 19 chromo-lithographic figures and 17 engravings. Cloth, $3.50. Philadelphia : Lea Brothers & Co. 1894.

Report of the Commissioner of Education for the Year 1890–91. Volumes I. and II. Washington : Government Printing Office. 1894.

An International System of Electro-therapeutics, for Students, General Practitioners and Specialists. By Horatio R. Bigelow, M. D., and Thirty-eight Associate Editors. Thoroughly illustrated. In one large royal octavo volume, 1,160 pages; extra cloth, $6.00 net ; sheep, $7.00 net ; half-Russia, $7.50 net. Philadelphia : The F. A. Davis Co., Publishers, 1914 and 1916 Cherry street.

